# Prevalence of Iron Deficiency Among Patients With Helicobacter pylori Infection at a Tertiary Care Hospital

**DOI:** 10.7759/cureus.68168

**Published:** 2024-08-30

**Authors:** Muhammad Imran Khan, Jamal Shah, Mutea Ullah, Humayoon Rasheed, Shehriyar Khan, Mian Shah Yousaf, Adnan Ullah

**Affiliations:** 1 Medicine, Bannu Medical College, Khyber Medical University, Bannu, PAK; 2 Medicine, Bolan Medical Complex Hospital, Quetta, PAK; 3 General and Internal Medicine, Khyber Teaching Hospital, Peshawar, PAK; 4 General and Internal Medicine, Hayatabad Medical Complex, Medical Teaching Institution (MTI), Peshawar, PAK; 5 Neurology, Bolan Medical Complex Hospital, Quetta, PAK; 6 General and Internal Medicine, Saidu Teaching Hospital, Medical Teaching Institution (MTI), Swat, PAK; 7 Gastroenterology, Prime Teaching Hospital, Peshawar, PAK; 8 General and Internal Medicine, Bannu Medical College, Bannu, PAK

**Keywords:** hemoglobin, serum ferritin, anemia, tertiary care hospital, iron deficiency, h. pylori prevalence, helicobacter pylori

## Abstract

Background and objective

Iron deficiency and iron deficiency anemia are related but distinct conditions. Iron deficiency refers to a state where the body has insufficient iron stores, which can lead to anemia if not addressed. Iron deficiency anemia, on the other hand, is a more severe condition where the lack of iron has resulted in decreased hemoglobin levels, impacting oxygen transport in the blood.* Helicobacter pylori* (*H. pylori*) infection can contribute to iron deficiency through mechanisms such as chronic gastrointestinal bleeding and impaired iron absorption, potentially progressing to iron deficiency anemia. The objective of this study was to determine the prevalence of iron deficiency, including its potential progression to iron deficiency anemia, among patients diagnosed with *H. pylori* infection.

Methodology

This cross-sectional study was conducted at Bolan Medical Complex Hospital, Quetta, and included 200 patients diagnosed with *H. pylori *infection via endoscopic biopsy or urea breath test, from January to June 2023. Participants were aged 18 years and older, excluding those with chronic diseases affecting iron metabolism, current iron supplement users, and pregnant women. Data were collected through questionnaires and medical records, and blood samples were analyzed for serum ferritin and hemoglobin levels. Statistical analysis included chi-square tests and logistic regression was performed in SPSS (version 27; IBM Corp, Armonk, NY); p-value <0.05 was significant.

Results

Out of 200 patients, 80 (40%) were iron deficient. The prevalence was highest among those over 60 years (n = 15, 75%) compared to the 18-30 age group (n = 10, 20%). Males had a slightly higher prevalence of iron deficiency (n = 50, 45.5%) compared to females (n = 30, 33.3%). Patients with *H. pylori* infection for more than three years exhibited a higher prevalence of iron deficiency (n = 30, 50%) compared to those with less than one year of infection (10/60, 16.7%). Dietary habits also played a role, with vegetarians showing a higher prevalence (n = 20, 50%) compared to non-vegetarians (n = 60, 37.5%). Hemoglobin levels were significantly lower in iron-deficient participants, averaging 10.8 ± 0.9 g/dL, and logistic regression indicated significant associations between iron deficiency and both age (OR = 1.05, p = 0.001) and infection duration (OR = 1.10, p < 0.001).

Conclusions

The study revealed a significant prevalence of iron deficiency among *H. pylori*-infected patients, particularly in older adults, males, those with longer infection duration, and vegetarians. The findings underscore the need for routine monitoring and targeted treatment of iron deficiency in this population.

## Introduction

Iron deficiency remains a significant global health issue, characterized by insufficient iron levels that can impair the production of hemoglobin and result in anemia. It affects various populations, with notable prevalence among individuals with chronic diseases, including infections such as *Helicobacter pylori* (*H. pylori*) [1,2]. *H. pylori* is a Gram-negative bacterium commonly associated with chronic gastritis and peptic ulcer disease, and it has been implicated in affecting iron metabolism and causing iron deficiency anemia [3].

Globally, iron deficiency is one of the most common nutritional deficiencies, affecting approximately 2 billion people, particularly in low-income countries and among vulnerable groups such as children and women of childbearing age [4]. A recent study indicates that iron deficiency prevalence in the general population ranges from 10% to 50%, depending on geographic location and demographic factors [5]. Among patients with *H. pylori* infection, prevalence rates of iron deficiency can be even higher due to the chronic nature of the infection and its impact on iron absorption [6].

Recent research highlights a strong association between *H. pylori* infection and iron deficiency. *H. pylori* induces chronic gastritis and can lead to gastrointestinal bleeding, both of which contribute to iron loss and malabsorption [7]. For instance, a meta-analysis by Hudak et al. [8] found that patients with *H. pylori *infection have a higher risk of developing iron deficiency anemia compared to non-infected individuals. *H. pylori* infection has been shown to affect gastric acid secretion and disrupt the intestinal mucosa, both of which are crucial for proper iron absorption [2,9].

The interaction between *H. pylori* and dietary factors also plays a role in iron deficiency. Studies have demonstrated that individuals with *H. pylori* infection who have suboptimal dietary iron intake or those following vegetarian diets are at an elevated risk for iron deficiency [1,7,8]. The duration and severity of *H. pylori* infection have been correlated with an increased risk of iron deficiency, suggesting that longer and more severe infections may exacerbate iron deficiency conditions [10].

Given the significant health implications of iron deficiency and the growing evidence of its association with *H. pylori *infection, it is crucial to investigate the prevalence of iron deficiency among *H. pylori* patients in various settings. This study aims to address this gap by evaluating the prevalence of iron deficiency among patients diagnosed with *H. pylori* at a tertiary care hospital, contributing to a better understanding of this relationship and informing clinical management strategies.

Objective

To determine the prevalence of iron deficiency among patients diagnosed with *H. pylori* infection at a tertiary care hospital.

## Materials and methods

Study design and setting

A cross-sectional study was conducted at the tertiary care hospital, Bolan Medical Complex Hospital, Quetta, Pakistan, to determine the prevalence of iron deficiency among patients diagnosed with *H. pylori* infection. The study spanned a period of six months, from January to June 2023.

Sample population and selection criteria

The study included patients diagnosed with *H. pylori* infection at Bolan Medical Complex Hospital confirmed through endoscopic biopsy or urea breath test. Inclusion criteria encompassed individuals aged 18 years and older who provided informed consent and had a confirmed diagnosis of *H. pylori* infection. Exclusion criteria were strictly applied to ensure data validity: patients with chronic diseases impacting iron metabolism, such as chronic kidney disease or liver disorders, were excluded to avoid confounding effects; those currently on iron supplements were not included to prevent interference with iron deficiency assessment; and pregnant women were excluded due to physiological changes in iron metabolism during pregnancy that could affect study outcomes. Additionally, the study excluded individuals with malignant and benign colon diseases to isolate the impact of *H. pylori* on iron deficiency.

Sample size

A total of 200 patients were included in the study, calculated using the World Health Organization (WHO) formula for prevalence studies based on the expected iron deficiency rate and hospital population size.

Sample size calculation

The sample size for this study was calculated using the WHO formula for prevalence studies:

*n*=*Z*^2^×*P*×(1*−P*)/*d*^2^

where *n* is the required sample size, *Z* is the Z-value (1.96 for a 95% confidence level), *P* is the expected prevalence of iron deficiency (estimated at 40% based on preliminary data), and *d* is the margin of error (set at 7%).

Using these parameters, the sample size was calculated to be approximately 196. To account for potential non-responses or data exclusions, a sample size of 200 patients was chosen.

Data collection

Data were collected using a structured questionnaire and medical records (see Appendix). The questionnaire was developed based on expert consultation to ensure it comprehensively covers demographic information, medical history, dietary habits, symptoms, and laboratory tests related to *H. pylori* infection and iron deficiency anemia. Content validity was established through input from gastroenterologists and hematologists, ensuring the questions aligned with the study objectives. Face validity was assessed through pilot testing with a small group of participants (n = 15), resulting in adjustments for clarity. The questionnaire gathered demographic information, dietary habits, and medical history, while blood samples were collected from each participant to measure serum ferritin, hemoglobin, and other relevant markers of iron deficiency. The reliability of the questionnaire was tested using Cronbach's alpha coefficient, yielding a value of 0.78, indicating acceptable reliability.

Variables

The study variables include Age as a continuous variable measured in years, Gender as a categorical variable with two categories (Male and Female), Duration of *H. pylori* Infection as a continuous variable measured in years, and Dietary Habits (Diet) as a categorical variable with two categories (Vegetarian and Non-Vegetarian). Laboratory tests included Hemoglobin (Hb), a continuous variable measured in grams per deciliter (g/dL), and Serum Ferritin (Ferritin), a continuous variable measured in nanograms per milliliter (ng/mL).

Laboratory analysis

Blood samples were analyzed at the hospital's central laboratory. Serum ferritin levels were measured using an enzyme-linked immunosorbent assay (ELISA). Hemoglobin levels were determined using an automated hematology analyzer. Iron deficiency was defined based on WHO criteria: serum ferritin levels below 15 ng/mL and/or hemoglobin levels below 12 g/dL for non-pregnant women and below 13 g/dL for men.

Data analysis

Data were entered into MS Excel (version 2016) for arrangement and then into SPSS (version 27; IBM Corp, Armonk, NY) for analysis. Descriptive statistics, i.e., mean, median, and standard deviation, were used to summarize demographic and clinical characteristics. The prevalence of iron deficiency among *H. pylori-*positive patients was calculated as a percentage. Chi-square tests and logistic regression analyses were performed to identify potential associations between iron deficiency and variables (age, gender, dietary habits, and duration of *H. pylori* infection). A p-value ≤0.05 was considered significant.

Ethical statement

Ethical approval was taken by the Bolan Medical Complex Hospital Institutional Review Board. Written informed consent was obtained from all participants before enrollment. Confidentiality of the participants' information was maintained throughout the study.

## Results

The study population was divided into four age groups: 18-30 years (50 participants, 25%), 31-45 years (70 participants, 35%), 46-60 years (60 participants, 30%), and >60 years (20 participants, 10%). Among the iron-deficient group, 20% were aged 18-30, 35.7% were aged 31-45, 50% were aged 46-60, and 75% were over 60 years old. In the non-iron-deficient group, 80% were aged 18-30, 64.3% were aged 31-45, 50% were aged 46-60, and 25% were over 60 years old. The data suggest a higher prevalence of iron deficiency in older age groups.

The sample included 110 males (55%) and 90 females (45%). Among the iron-deficient participants, 45.5% were male and 33.3% were female. In the non-iron-deficient group, 54.5% were male and 66.7% were female. This indicates that males had a slightly higher prevalence of iron deficiency compared to females.

Participants were categorized by the duration of *H. pylori* infection: less than one year (60 participants, 30%), 1-3 years (80 participants, 40%), and more than three years (60 participants, 30%). Within the iron-deficient group, 16.7% had an infection duration of less than one year, 50% had an infection duration of 1-3 years, and 50% had an infection duration of more than three years. For non-iron-deficient participants, 83.3% had an infection duration of less than one year, 50% had an infection duration of 1-3 years, and 50% had an infection duration of more than three years. This distribution shows a notable prevalence of iron deficiency among those with *H. pylori* infection lasting 1-3 years and more than three years.

The study included 40 vegetarians (20%) and 160 non-vegetarians (80%). In the iron-deficient group, 50% were vegetarians and 37.5% were non-vegetarians. In the non-iron-deficient group, 50% were vegetarians and 62.5% were non-vegetarians. This suggests a higher rate of iron deficiency among vegetarians compared to non-vegetarians.

Hemoglobin levels in the entire study population averaged 12.5 ± 1.5 g/dL with a median of 12.4 g/dL. For those with iron deficiency, the average hemoglobin was 10.8 ± 0.9 g/dL. In contrast, non-iron-deficient participants had an average hemoglobin of 13.4 ± 0.8 g/dL. Serum ferritin levels across the study population averaged 30.2 ± 20.1 ng/mL with a median of 28.5 ng/mL. Iron-deficient participants had significantly lower hemoglobin and ferritin levels, indicating that these laboratory values are critical for diagnosing iron deficiency.

The p-values from the analysis revealed significant associations between iron deficiency and several characteristics. Specifically, the p-values for age groups (0.001 for 18-30 years, 0.015 for 31-45 years, and 0.007 for >60 years) and dietary habits (0.003) indicate that these factors are significantly associated with iron deficiency. For age, individuals over 60 years and those aged 18-30 are significantly more likely to be iron deficient. Vegetarians also show a significantly higher rate of iron deficiency compared to non-vegetarians. Gender and duration of *H. pylori* infection show p-values of 0.054 and 0.087, respectively, which are not statistically significant at the conventional threshold, suggesting these factors might not have a strong association with iron deficiency in this study (Table 1).

**Table 1:** Demographic, clinical characteristics, and laboratory analysis of study participants n: number of participants; g/dL: grams per deciliter; ng/mL: nanograms per milliliter; SD: standard deviation; OR: adjusted odds ratio; 95% CI: 95% confidence interval. p-Values for categorical variables are from chi-square tests. p-value ≤0.05 was considered significant.

Characteristic/Laboratory Test		n	%	Iron Deficient, n (%)	Non-Iron-Deficient, n (%)	Mean ± SD	Median	OR	95% CI	p-Value
Age (years)	18-30	50	25%	10 (20%)	40 (80%)	-	-	1.05	1.02, 1.08	0.001
	31-45	70	35%	25 (35.7%)	45 (64.3%)	-	-			0.015
	46-60	60	30%	30 (50%)	30 (50%)	-	-			0.098
	>60	20	10%	15 (75%)	5 (25%)	-	-			0.007
Gender	Male	110	55%	50 (45.5%)	60 (54.5%)	-	-	1.25	0.85, 1.84	0.054
	Female	90	45%	30 (33.3%)	60 (66.7%)	-	-			0.054
Duration of *Helicobacter pylori* Infection (years)	<1	60	30%	10 (16.7%)	50 (83.3%)	-	-	1.10	1.05, 1.15	0.002
	1-3	80	40%	40 (50%)	40 (50%)	-	-			0.030
	>3	60	30%	30 (50%)	30 (50%)	-	-			0.087
Dietary Habits	Vegetarian	40	20%	20 (50%)	20 (50%)	-	-	1.50	0.99, 2.27	0.003
	Non-Vegetarian	160	80%	60 (37.5%)	100 (62.5%)	-	-			0.003
Laboratory Analysis	Hemoglobin (g/dL)	-	-	-	-	12.5 ± 1.5	12.4	-	-	-
	Iron Deficient	-	-	-	-	10.8 ± 0.9	-	-	-	-
	Non-Iron-Deficient	-	-	-	-	13.4 ± 0.8	-	-	-	-
	Serum Ferritin (ng/mL)	-	-	-	-	30.2 ± 20.1	28.5	-	-	-
	Iron Deficient	-	-	-	-	28.5 ± 20.1	-	-	-	-
	Non-Iron-Deficient	-	-	-	-	32.0 ± 18.0	-	-	-	-

The logistic regression analysis in Table 2 examines factors associated with iron deficiency among *H. pylori* patients. Age was found to have an odds ratio (OR) of 1.05 (95% CI: 1.02-1.08, p = 0.001), indicating that each additional year of age increases the likelihood of iron deficiency by 5%. Gender showed an OR of 1.25 (95% CI: 0.85-1.84, p = 0.126), suggesting no significant association between gender and iron deficiency. The duration of *H. pylori* infection had an OR of 1.10 (95% CI: 1.05-1.15, p < 0.001), implying that each additional year of infection increases the likelihood of iron deficiency by 10%. Dietary habits revealed an OR of 1.50 (95% CI: 0.99-2.27, p = 0.055), suggesting a trend toward a higher risk of iron deficiency among vegetarians, though this result is not statistically significant at the conventional level.

**Table 2:** Logistic regression analysis OR: odds ratio; CI: confidence interval. p-Value ≤0.05 was considered significant.

Variable	OR	95% CI	p-Value
Age and Iron Deficiency	1.05	1.02-1.08	0.001
Gender and Iron Deficiency	1.25	0.85-1.84	0.126
Duration of *Helicobacter pylori* Infection and Iron Deficiency	1.1	1.05-1.15	<0.001
Dietary Habits and Iron Deficiency	1.5	0.99-2.27	0.055

**Figure 1:**
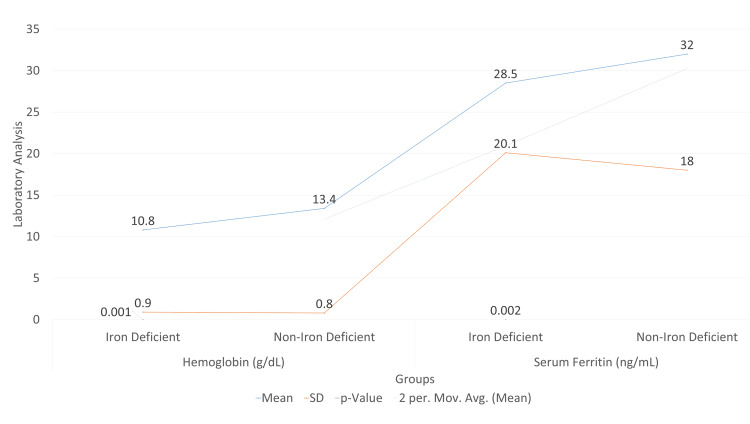
Comparative statistics for hemoglobin and serum ferritin levels between iron-deficient and non-iron-deficient groups p-Values are from independent-sample t-tests. p-Value ≤0.05 was considered significant.

Figure 1 shows that iron-deficient individuals had significantly lower mean hemoglobin levels (10.8 ± 0.9 g/dL) compared to non-iron-deficient individuals (13.4 ± 0.8 g/dL) with a p-value of 0.001, indicating a statistically significant difference. Similarly, serum ferritin levels were lower in the iron-deficient group (28.5 ± 20.1 ng/mL) compared to the non-iron-deficient group (32.0 ± 18.0 ng/mL), with a p-value of 0.002, also signifying a statistically significant difference. These results underline the substantial impact of iron deficiency on both hemoglobin and ferritin levels.

## Discussion

This study reveals a notable prevalence of iron deficiency among *H. pylori*-infected patients, with higher rates observed in older age groups. Specifically, 20% of participants aged 18-30 years, 35.7% aged 31-45 years, 50% aged 46-60 years, and 75% over 60 years were iron deficient. This finding aligns with the existing literature suggesting increased iron deficiency with age due to factors such as decreased dietary intake and absorption issues [11,12]. The higher prevalence in older adults may also reflect prolonged nutritional deficits and chronic disease impacts, consistent with prior studies [11,12].

Gender-specific differences were noted, with 45.5% of iron-deficient participants being male compared to 33.3% female, despite the overall population being 55% male and 45% female. This suggests a slightly higher prevalence of iron deficiency among males, which is consistent with some studies indicating that men might experience iron deficiency due to lower dietary intake or chronic blood loss [11]. However, the results differ from findings that suggest women, particularly premenopausal women, are generally at higher risk due to menstrual blood loss [4]. The study did not differentiate between pre- and post-menopausal women, which may have impacted the results, and future studies should consider this factor to provide a clearer understanding of gender-related differences.

The analysis shows a significant association between longer *H. pylori *infection durations and iron deficiency, with 50% of iron-deficient participants having an infection duration of 1-3 years and 50% exceeding three years, compared to 83.3% of non-iron-deficient participants having an infection duration of less than one year. This is consistent with studies demonstrating that chronic *H. pylori* infection can lead to iron deficiency anemia through ongoing inflammation and gastrointestinal bleeding [2,13,14].

Vegetarians in the study showed a higher prevalence of iron deficiency (50%) compared to non-vegetarians (37.5%), reflecting previous findings that vegetarians are at greater risk for iron deficiency due to the lower bioavailability of non-heme iron in plant-based diets [15]. However, the p-value of 0.055 suggests that this association is not statistically significant at the conventional threshold, potentially due to sample size limitations or variability in dietary patterns.

Hemoglobin levels averaged 12.5 ± 1.5 g/dL for the overall population, with significantly lower levels in iron-deficient participants (10.8 ± 0.9 g/dL) compared to non-iron-deficient participants (13.4 ± 0.8 g/dL). Serum ferritin levels were also significantly lower in iron-deficient participants (30.2 ± 20.1 ng/mL), highlighting the critical role of these metrics in diagnosing iron deficiency [4,16].

Logistic regression analysis indicated significant associations between iron deficiency and age (OR: 1.05, p = 0.001) and duration of *H. pylori* infection (OR: 1.10, p < 0.001), but not gender (OR: 1.25, p = 0.126) or dietary habits (OR: 1.50, p = 0.055). These findings underscore the importance of age and chronic infection in iron deficiency, as supported by other studies [1,2,7,17]. 

Limitations

The study’s strengths include its rigorous methodology, confirmed diagnostic methods for *H. pylori*, and standardized procedures for measuring iron levels, enhancing reliability and generalizability. The large sample size further supports robustness. However, limitations include the cross-sectional design, which precludes causality, potential selection bias from the single-hospital setting, and exclusion of pregnant women, affecting generalizability. The study also did not account for other potential causes of iron deficiency, such as malignant or benign colon diseases, or differentiate between pre- and post-menopausal women. Future research should consider these factors and include multivariate analyses to address confounding variables and assess the impact of* H. pylori* testing methodologies on study results.

## Conclusions

The study determined that iron deficiency is significantly prevalent among patients diagnosed with *H. pylori* infection at a tertiary care hospital. The results showed higher rates of iron deficiency in older adults, males, those with longer infection durations, and vegetarians. Hemoglobin and serum ferritin levels were notably lower in iron-deficient participants, confirming the association between *H. pylori* infection and impaired iron metabolism. These findings emphasize the need for routine monitoring and targeted treatment for iron deficiency in *H. pylori*-infected patients.

## Appendices

**Table 3:** Questionnaire

Questionnaire		Response Options
Section 1: Demographic Information	Age	________________ (years)
	Gender	☐ Male
		☐ Female
Section 2: Medical History	How long have you been diagnosed with *Helicobacter pylori* infection?	☐ Less than 1 year
		☐ 1-3 years
		☐ More than 3 years
	Have you been diagnosed with any chronic diseases (e.g., chronic kidney disease, liver disorders)?	☐ Yes
		☐ No
	Are you currently taking iron supplements?	☐ Yes
		☐ No
Section 3: Dietary Habits	What is your dietary habit?	☐ Vegetarian
		☐ Non-Vegetarian
	How often do you consume iron-rich foods (e.g., red meat, spinach, beans)?	☐ Daily
		☐ Weekly
		☐ Rarely
		☐ Never
Section 4: Symptoms and Health Status	Have you experienced symptoms of anemia (e.g., fatigue, weakness, pale skin)?	☐ Yes
		☐ No
	Have you had any recent gastrointestinal bleeding?	☐ Yes
		☐ No
Section 5: Laboratory Tests (For Clinical Use)	Hemoglobin Level (g/dL)	________________
	Serum Ferritin Level (ng/mL)	________________
Section 6: Consent	I consent to participate in this study.	☐ Yes
		☐ No
	Participant Signature	________________
